# Supplementary material for: Changes in Vitamin D Status in Korean Adults during the COVID-19 Pandemic
Source: Nutrients. 2022 Nov 17;14(22):4863. doi: 10.3390/nu14224863 (PMC9696842; doi:10.3390/nu14224863)
Supplement: Supplementary file 1 [file nutrients-14-04863-s001.zip › nutrients-2007744-supplementary.pdf]

Figure S1. Distribution of physical activity by gender before and after the COVID-19 pandemic. (A) Before COVID-19 lockdown; (B) During COVID-19 lockdown.

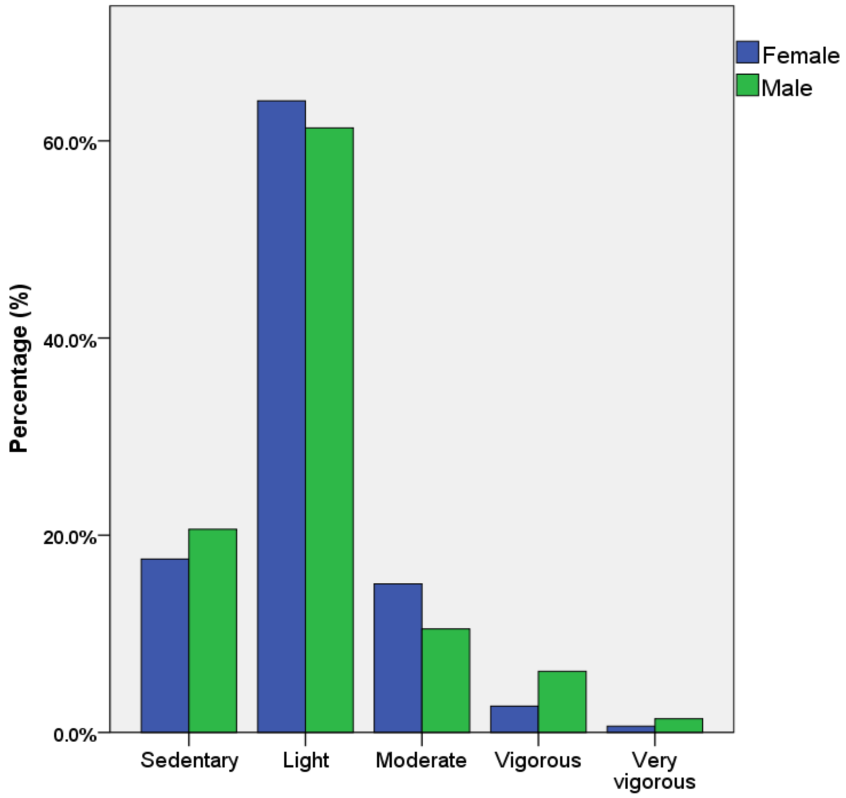

(A) Before COVID-19 Lockdown

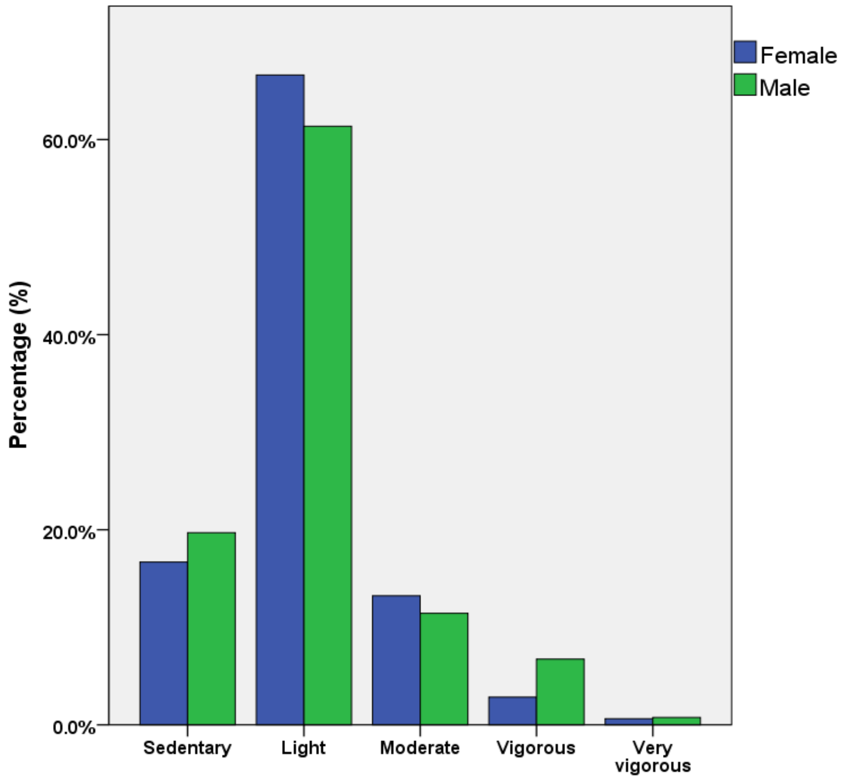

(B) During COVID-19 Lockdown

Figure S2. Distribution of examination time by gender before and after the COVID-19 pandemic. (A) Before COVID-19 lockdown; (B) During COVID-19 lockdown.

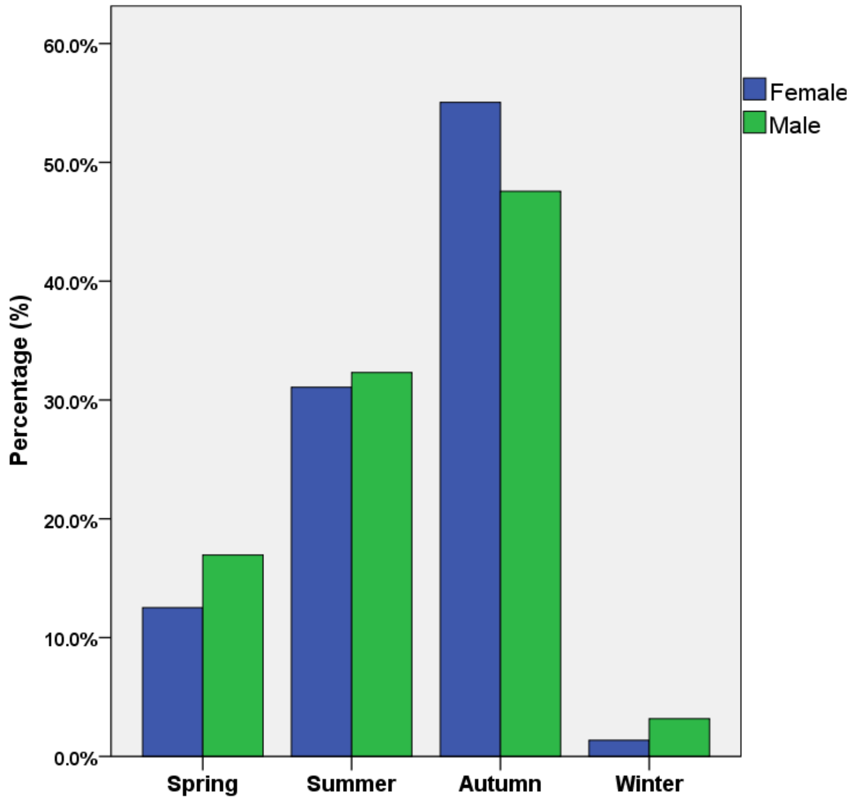

(A) Before COVID-19 Lockdown

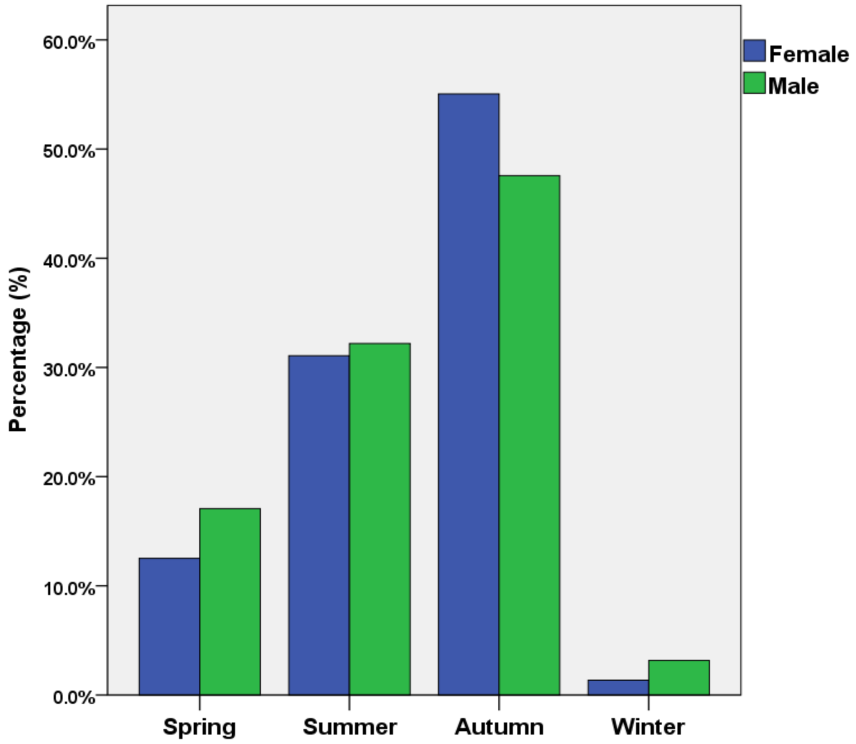

(B) During COVID-19 Lockdown
